# Design of Path-Planning System for Interventional Thermal Ablation of Liver Tumors Based on CT Images

**DOI:** 10.3390/s24113537

**Published:** 2024-05-30

**Authors:** Ziwei Song, Feifei Ding, Weiwei Wu, Zhuhuang Zhou, Shuicai Wu

**Affiliations:** 1 Department of Biomedical Engineering, College of Chemistry and Life Sciences, Beijing University of Technology, Beijing 100124, China; songziwei1121@163.com (Z.S.); dffest@foxmail.com (F.D.); zhouzh@bjut.edu.cn (Z.Z.); wushuicai@bjut.edu.cn (S.W.); 2 College of Biomedical Engineering, Capital Medical University, Beijing 100069, China

**Keywords:** thermal ablation of tumors, surgical planning, weighted summation, Pareto optimality, system design

## Abstract

Objective: Aiming at the shortcomings of artificial surgical path planning for the thermal ablation of liver tumors, such as the time-consuming and labor-consuming process, and relying heavily on doctors’ puncture experience, an automatic path-planning system for thermal ablation of liver tumors based on CT images is designed and implemented. Methods: The system mainly includes three modules: image segmentation and three-dimensional reconstruction, automatic surgical path planning, and image information management. Through organ segmentation and three- dimensional reconstruction based on CT images, the personalized abdominal spatial anatomical structure of patients is obtained, which is convenient for surgical path planning. The weighted summation method based on clinical constraints and the concept of Pareto optimality are used to solve the multi-objective optimization problem, screen the optimal needle entry path, and realize the automatic planning of the thermal ablation path. The image information database was established to store the information related to the surgical path. Results: In the discussion with clinicians, more than 78% of the paths generated by the planning system were considered to be effective, and the efficiency of system path planning is higher than doctors’ planning efficiency. Conclusion: After improvement, the system can be used for the planning of the thermal ablation path of a liver tumor and has certain clinical application value.

## 1. Introduction

Primary liver cancer is the seventh most common cancer in the world, seriously endangering human life and health [1]. With the advancement of medical imaging technology, the detectability of early-stage liver cancer is being improved and the use of local thermal ablation technology to treat liver cancer is also being significantly developed [2].

Thermal ablation is a treatment method that uses a high temperature to treat tumor tissue and destroys the biological structure of tumor cells. This technology achieves the purpose of ablating tumor tissue by transmitting radio frequency, microwave, and other energy into tumor tissue, causing local heating of the tissue and necrosis and coagulation [3,4]. Compared with traditional surgical resection, thermal ablation has the advantages of less trauma, faster recovery, easy operation, and repeatable operation. It is especially suitable for patients with small liver tumors that cannot be surgically removed or patients with high surgical risks. Therefore, thermal ablation technology is widely used in the treatment of liver tumors and has achieved good clinical results [5,6,7,8].

The treatment process of liver tumor thermal ablation can be divided into three stages: preoperative planning, intraoperative implementation (puncture), and postoperative evaluation. Preoperative planning is the first and most critical step in treatment. There are two tasks that need to be completed. The first task is to determine the anatomical structures of the patient’s abdominal cavity and the relative position of the liver tumor. This relies on image processing techniques to segment and reconstruct all the structures of the abdominal cavity. The second task is to plan the path and treatment parameters of the ablation needle (such as ablation power and duration). Especially ensuring precise intervention of the ablation needle into the central target of the tumor is crucial for thermal ablation therapy [9,10,11,12]. However, currently, both path planning (the optimal path for the ablation needle to intervene) and puncture guidance (real-time monitoring of the position of the ablation needle in the body) rely on the personal experience of clinicians. The planning process is not only time-consuming and laborious, but also presents challenges in accommodating multiple clinical guidelines. During the surgery, the doctor holds the ablation needle and makes a direct puncture. Throughout the operation, the doctor also needs to make real-time adjustments and judgments based on factors such as the location and size of the tumor, which increases the complexity and risks of the surgery and also requires higher professional skills from the doctor.

In recent years, with the rapid development of image processing and visualization technology, the use of computer-assisted automatic or semi-automatic surgical path planning has become a research hotspot in the field of thermal ablation therapy [13]. Liu et al. [14] developed a computer-aided 3D visualization preoperative treatment planning system that can visualize the spatial relationship between tumors and surrounding structures in a 3D manner, calculate the distance between tumors and important structures or organs in the surrounding area, and provide the minimum and optimal insertion times, as well as the needle and other treatment parameters required in the simulated environment. Ren et al. [15] developed a systematic approach for needle-based ablation placement tasks, ranging from preoperative planning algorithms to intraoperative execution platforms. This planning system combines clinical constraints on ablation and trajectory using multi-objective optimization formulas, including integer programming-based optimal path selection and ablation coverage optimization. Li et al. [16] proposed a multi-objective optimization independent GPU-based multi-puncture planning method for liver tumor thermal ablation surgery. Based on this method, preprocessing planning software was developed that visualized the planning results in a 3D scene using DICOM images as the input. Alice et al. [17] developed an easily scalable ablation surgery planning platform based on the open-source software 3D Slicer-4.10.2, which provides some common functions, including segmentation algorithms and dose calculations. The system can display thermal dose distribution on 2D image slices and 3D structures, support repeated editing and viewing of treatment plans, and assist doctors in surgical path planning. Franz et al. [18] designed a multi-needle ablation planning system for larger tumors based on the open-source framework MITK (the Medical Imaging Interaction Toolkit), focusing on the number and placement of ablation needles. Retrospective validation studies showed that system-planned paths were comparable to manual planning by clinicians in terms of the required number of ablation zones. However, the ablation-planning systems mentioned above do not fully consider clinical treatment needs and are mainly semi-automatic planning. Although they provide certain visualization solutions, the planning of complex cases is still difficult.

To this end, we propose an automatic planning method for a liver tumor thermal ablation puncture path based on CT imaging, taking into account clinical treatment needs and treatment processes. By summarizing clinical treatment guidelines and objectives and quantifying them into multiple objective functions, the path-planning problem is essentially transformed into a multi-objective optimization problem. We introduced weighted summation and the Pareto optimality theory for multi-objective decision-making problems and developed an automatic path-planning system for liver tumor interventional thermal ablation surgery based on CT imaging, using computers to automatically plan the optimal path for reference and selection. The purpose is to provide an automated solution that reduces the difficulty of ablation surgical path planning, improves the objectivity and efficiency of surgical planning, and can guide or assist doctors in clinical treatment. It has certain research significance and clinical value.

## 2. Methods

The design of the software system refers to clinical needs and treatment processes. Firstly, there needs to be a visual interface that is easy to operate, making it convenient for doctors to browse and analyze images. Secondly, the system needs to include certain image processing functions, such as image segmentation and 3D reconstruction, to make it easier for doctors to determine the anatomical position of the liver, tumors, blood vessels, and other important organs and tissues in the patient’s abdominal space, replacing the complex and laborious process of relying on experience and imagination, and based on this, plan the surgical path. In addition, the system should include a surgical path-planning module that can provide automatic or semi-automatic surgical path-planning solutions and display path-related parameters, allowing doctors to interact and revise paths as needed. Finally, it is necessary to include a data information storage module that can store, view, and print patient-related information, image data, and surgical planning details.

**Figure 1:**
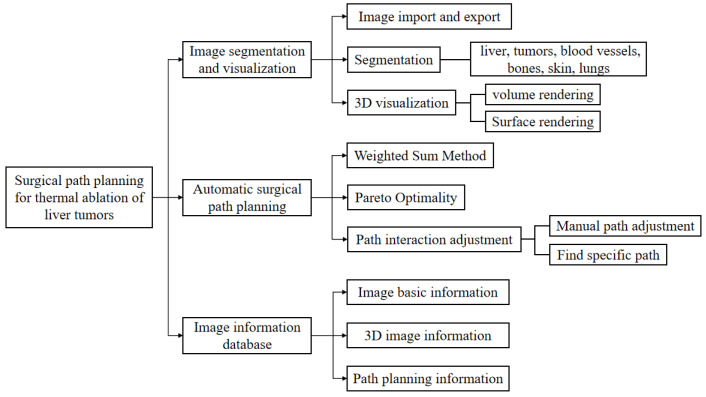
Functional module of liver tumor thermal ablation surgery path-planning system.

Based on the requirements above, the software system was designed and implemented using the Qt class library in the C++ language development scenario of Visual Studio-2017. The system includes three functional modules: image segmentation and a 3D visualization module, a surgical path planning module, and an image data management module, as shown in Figure 1. The segmentation and registration toolkit ITK-4.13 (Insight Toolkit) was used to implement related image processing algorithms, the visualization toolkit VTK-8.2 (The Visualization Toolkit) was used to implement a visual display, and the SQLite lightweight database was used for data storage. The system provides a complete set of automated solutions (automatic image segmentation, one-click 3D reconstruction, automatic surgical path planning), and supports users to interactively set the parameters according to their needs.

### 2.1. Image Segmentation and 3D Visualization Module

**Figure 2:**
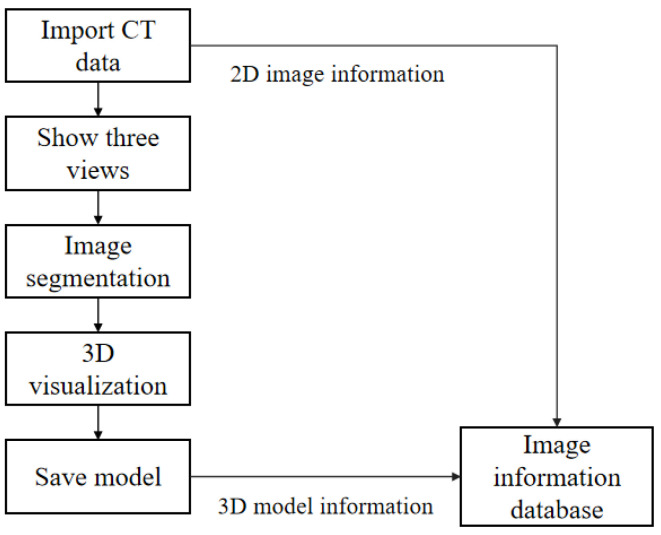
Image segmentation and 3D visualization module usage flow chart.

The segmentation and 3D visualization module includes functions such as displaying a three-dimensional view of CT sequences, parsing DICOM image information, automatic segmentation of key abdominal organs, volume rendering based on ray projection and surface rendering and reconstruction based on moving cubes, and saving a 3D STL model. The usage process of this module is shown in Figure 2. Firstly, the patient’s CT images are imported into the system, which can display the coronal, sagittal, and cross-sectional images in three views, making it easier for doctors to browse the images. Secondly, the system integrates automatic segmentation algorithms for the liver, tumors, blood vessels, bones, skin, and lungs, and only needs to set the path for saving segmentation files to complete organ segmentation [19,20,21]. Finally, the 3D model can be saved as an STL-format file, and the solid model can be 3D printed.

For the segmentation of the liver and liver tumors, a novel automatic segmentation model based on the 3D UNet deep neural network incorporating the latest attention mechanism block and structural reparameterized residual blocks is proposed [19,20]. The attention block helps to capture multi-scale global contextual information. The residual block improves the expressive power of features and the capture ability of the network through extended grouping processing and step-by-step fusion strategies. The segmentation methods of liver vessels, skin, bone, and lung integrate the earlier research work of the laboratory [21]. A new method of 3D automatic segmentation of CT images based on vascular enhancement and fuzzy connection degree is adopted for liver vessels. It includes an improved vascular enhancement filtering algorithm and an improved fuzzy connection degree segmentation algorithm, whose core lies in the following: using the improved vascular enhancement response as the input response of the fuzzy affinity function in order to improve the segmentation accuracy of the hepatic vascular segmentation method in CT images, especially in low-contrast CT images. The three-dimensional automatic segmentation of abdominal skin is performed using the empirical threshold segmentation method and morphological transformation. The multi-threshold Otsu algorithm, Euclidean distance transform, reverse normalization of gray intensity, and weighting operation are used in the 3D automatic bone segmentation. The lungs are segmented using the experience threshold.

### 2.2. Surgical Path-Planning Module

**Figure 3:**
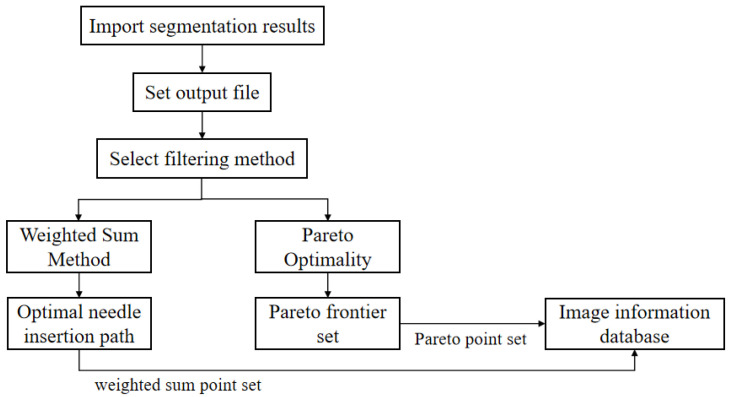
The usage flowchart of the automatic surgical path-planning module.

The surgical path-planning module is designed based on multiple constraints of clinical treatment, including two path-selection methods: the weighted summation method and the Pareto optimal method, which supports interactive adjustment of the surgical path. The usage process of this module is shown in Figure 3. Firstly, it is necessary to set the input and output files, that is, import the segmented key tissue organs and set the output file path. Secondly, two path-selection methods are available. The weighted sum method can output the optimal puncture path according to the score, and the Pareto optimality can output a set of Pareto frontier path points for reference.

#### 2.2.1. Algorithm Principle

In tumor thermal ablation therapy, the surgical pathway can be abstracted as the connecting line segment between the needle insertion point on the surface of the body and the liver tumor target. The surgical path-planning method based on clinical constraints has been a hot topic in computer-assisted surgical path planning in recent years. Its principle is to quantify clinical treatment needs and goals into multiple constraints and abstract the path planning problem into a multi-objective optimization problem. At present, relevant clinical constraints can be divided into hard constraints and soft constraints. Hard constraints refer to the clinical guidelines that surgical path planning must abide by (i.e., the puncture path should avoid contact with key abdominal structures, the length of puncture path must be strictly less than the ablation needle length, and the angle between the puncture path and the liver capsule should not be less than the clinical threshold). If these constraints are violated, the surgical path planning will fail. The set of body surface areas that satisfy all the hard constraints is the feasible needle insertion regions, and the connection between any voxel point in this area and the tumor center of mass can be used as a surgical path. Soft constraints refer to the clinical goals of surgical path planning (such as the distance between the puncture path and the key structures of the abdominal cavity should be as far apart as possible, the length of the puncture path should be as short as possible, and the angle between the puncture path and the liver capsule should be as large as possible, etc.). The higher the degree of compliance with them, the better the path. The priority of each voxel point in the feasible areas as the puncture needle target is obtained by applying a combination of soft constraints [22,23].

This system integrates two priority setting schemes for needle target points: one is a weighted sum method based on setting weights, and the other is a path-selection method based on the Pareto concept.

#### 2.2.2. Weighted Sum

According to the degree of compliance of each voxel point in the feasible areas with the soft constraint conditions, its score value is linearly set as the priority evaluation method. For example, for the constraint “the distance between the puncture path and key abdominal structures should be as far apart as possible”, the distance between each voxel point in the insertion area and the risk organ is traversed, and the minimum value among these distance data is used as the actual value of the distance to the risk structure. After normalization, this value can be used as a candidate voxel point to evaluate the score of the soft constraint condition.

Set the weight for each soft constraint and use the sum of the products method of Formula (1) for weighting. Among them, $Ps_{1}-Ps_{3}$ are the score evaluation of the voxel point P under each soft constraint condition, $\alpha_{1}-\alpha_{3}$ are the weights of each soft constraint condition, and the obtained result is the fractional value of voxel point P as the target point for needle insertion. In this way, the score evaluation of each voxel point in the insertion area is established as a needle-injection target, and the system can recommend 2–3 better paths based on the scores for operators to choose.

(1) $$P_{score}=Ps_{1}*\alpha_{1}+Ps_{2}*\alpha_{2}+Ps_{3}*\alpha_{3}$$

#### 2.2.3. Pareto Optimality

Since there is no gold standard for setting the weight of surgical path planning, the weight coefficients set by different clinicians are often different. Therefore, although our system supports user-defined weight settings, the planning results may still contain errors caused by subjective factors. This is also the current controversy in path-planning algorithms based on weighted summation. Therefore, in addition to providing a weighted sum path-planning method, our system also provides a path-planning method based on Pareto optimality for users to choose.

**Figure 4:**
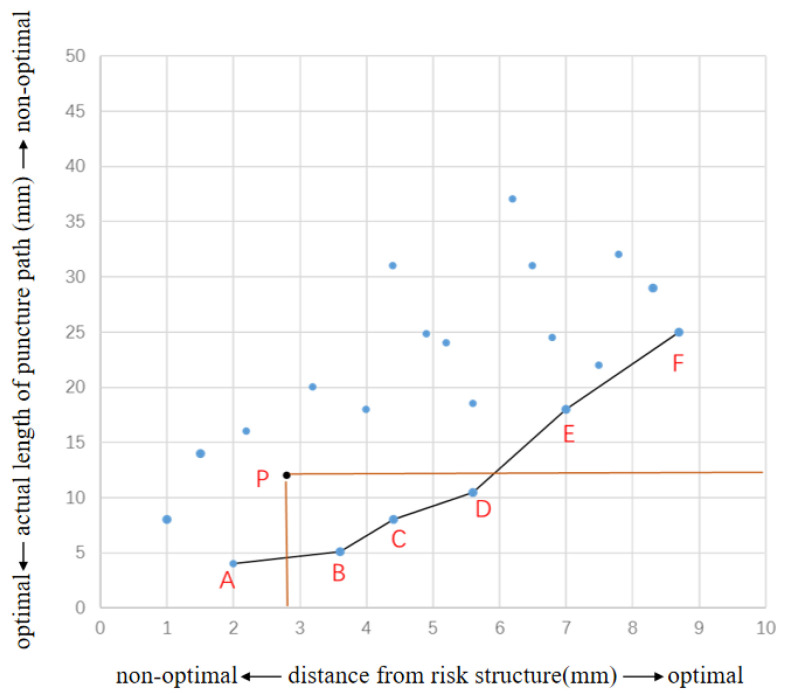
Pareto coordinate system constructed by “actual length of puncture path” and “distance to risk structure”.

Although semi-heuristic algorithms [24], genetic algorithms [25], clustering algorithms [26], and reinforcement learning [27] can handle multi-objective problems, they pose certain challenges in terms of implementation complexity and computational time. Pareto optimality, due to its flexibility, fairness, and effectiveness in solving complex problems, is now widely used in multi-objective optimization and is an ideal state for resource allocation. Our system uses multiple constraints of path planning as the objective function of Pareto optimization and introduces the concept of Pareto optimality for surgical path planning [28,29]. As shown in Figure 4, a Pareto coordinate system is constructed with the constraints of “actual length of puncture path” (vertical axis) and “distance to risk structure” (horizontal axis). The blue points in the coordinate system represent all the point sets within the feasible areas. The actual length of the puncture path needs to be as short as possible, so the smaller the value on the vertical axis, the better the path with that point as the puncture target. The distance between the puncture target and the risk structure needs to be as large as possible, so the larger the value on the horizontal axis, the better the path with this point as the puncture target. Taking point P in Figure 4 as an example, the vertical colored line represents the actual length of the puncture path with point P as the entry point, and the horizontal colored line represents the distance between point P as the entry point and the risk structure. In the coordinate system, there are three voxel points, B, C, and D, that satisfy the fact that the actual length of the puncture path is shorter than point P and the distance from the risk structure is larger than point P. Therefore, B, C, and D voxel points are better than point P. Point P is a point that can be optimized and is not a Pareto optimal point. There are no points in the coordinate system where both constraint values are better than points A−F, so points A−F belong to the Pareto optimality, and the set of points formed by them is called the Pareto front.

In addition, clinicians can also select a skin injection target (such as point P) based on the puncture experience. The system undergoes Pareto screening and outputs three points, B, C, and D, which are better than the clinician’s selected needle insertion point P. The needle insertion point can guide or assist clinicians in path planning.

All the clinical constraints are combined in pairs according to the rules above to establish a Pareto coordinate system, and the Pareto optimal points under each coordinate system are obtained, respectively. Finally, the intersection is calculated to obtain the Pareto frontier points set in the global context. Implement a method for path filtering that does not rely on weight settings.

### 2.3. Image Information Database Module

The image information database module can save patient information (patient name, age, and other related information obtained by parsing DICOM files), image information (basic image information, reconstructed organ volume, and other data), surgical planning parameter information (weighted path score information, Pareto frontier point set, etc.), etc., to the local database, making it convenient for operators to query, compare, and select.

## 3. Results

**Figure 5:**
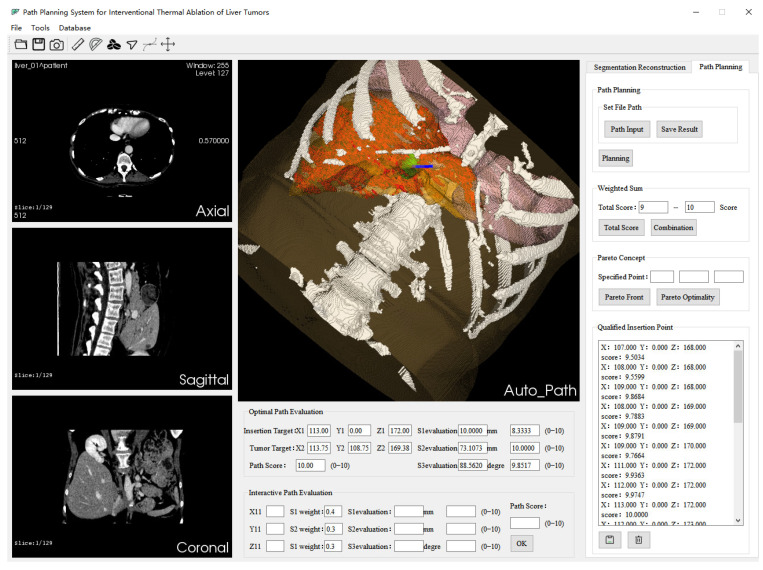
Surgical path-planning system interface.

Using our designed system for automatic surgical path planning, the system interface is shown in Figure 5. The system contains four image display windows. The three columns on the left are used for 3D display, and the center window is used to display the results of 3D organ reconstruction and the path-planning results (the green sphere represents the 3D reconstructed liver tumor, and the blue line segment represents the automatically planned optimal ablation needle puncture path). The bottom of the center window displays relevant information about the optimal path obtained by the “weighted sum method”, including the coordinates of the needle target point (X1,Y1,Z1), the coordinates of the tumor target point (X2,Y2,Z2), the absolute values of clinical constraints (the actual distance between the puncture path and the key structure S1, the actual length of the puncture path S2, the actual angle between the puncture path and the liver capsule S3), and the normalized relative value evaluation results. The weights of clinical constraints S1, S2, and S3 can be set according to the requirements. The path score is obtained by default based on the S1 weight of 0.3, S2 weight of 0.4, and S3 weight of 0.3. Its value ranges from 0 to 10, with 10 indicating the optimal path under the scoring criteria.

**Figure 6:**
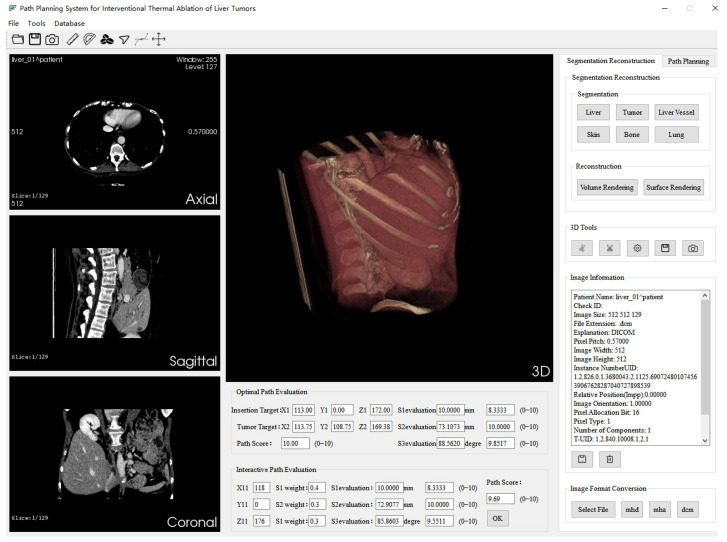
“Segmentation and Reconstruction” page of the surgical path-planning system.

**Figure 7:**
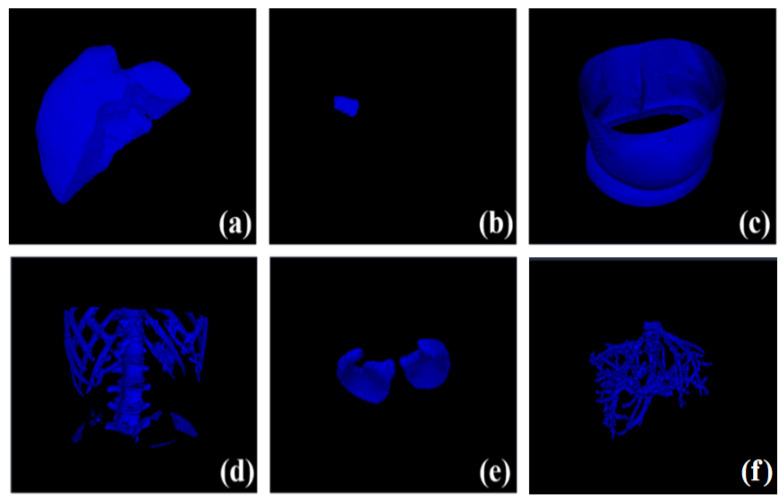
Isosurface rendering results of critical structures segmented in CT image by our system. (**a**–**f**) Volume rendering of segmented liver, liver tumor, skin, bone, lung, and liver vessel.

**Figure 8:**
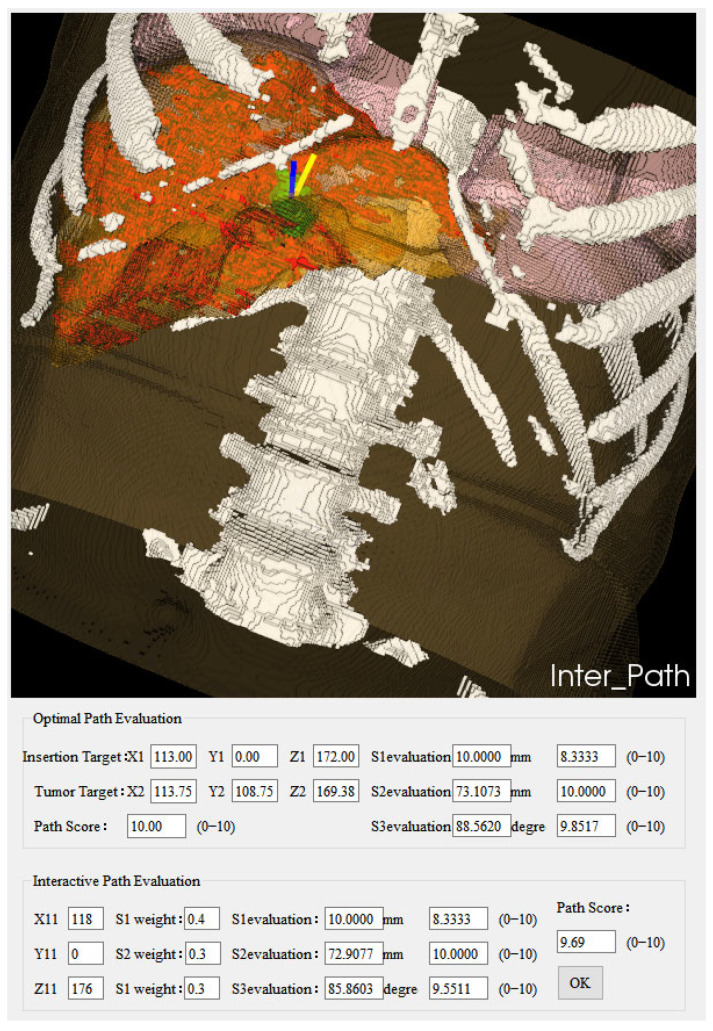
Interactively adjusted surgical path system interface. “Optimal Path Evaluation”: The planning parameters for the automatically planned ablation needle puncture path. “Optimal” refers to the needle target with the highest score evaluation within the feasible injection area. “Interactive Path Evaluation”: Specify a new puncture starting point coordinate to generate a new ablation needle puncture path, and display the planning parameters of the interactively adjusted ablation needle puncture path.

On the far-right of the system interface are switchable property pages, which are “Segmentation and Reconstruction” and “Path Planning”. The “Segmentation and Reconstruction” property page includes relevant implementation buttons for automatic segmentation of key structures of CT data, 3D reconstruction (volume rendering and surface rendering), and image information. Figure 6 illustrates the interface of the “Segmentation and Reconstruction” property page in the system, while Figure 7 showcases the isosurface rendering outcomes of the key structures within the CT images segmented by the system. The “Path Planning” property page contains relevant implementation buttons for path setting, the weighted sum method, and the Pareto optimality method. Below are other feasible reference needle target coordinate data displayed according to the path score. The system supports interactive adjustment and display of the puncture path. The path information manually added by the operator is displayed in the “Interactive Path Evaluation” box below. As shown in Figure 8, the yellow line segment is the adjusted puncture path, and the coordinates of the needle insertion target point are (X11,Y11,Z11).

Since there is no gold standard for puncture planning, we referred to other related studies [30] and invited two experienced clinicians engaged in liver tumor thermal ablation to evaluate the scientificity and effectiveness of the systematically generated puncture pathway. Ten three-dimensional CT images were selected from the publicly available dataset 3Dircadb, which include 18 liver tumors (diameter < 3 cm). Firstly, the two clinicians manually planned the path for these tumors, and then used this system for automatic path planning. The clinicians conducted a rationality comparison evaluation of the two, with evaluation criteria set at four categories: Excellent, Acceptable, Uncertain, and Unreasonable. The clinicians’ judgment and division of the four categories were mainly compared with the manually planned paths. The evaluation results are shown in Table 1. The manually interactive adjustment was performed on the puncture paths that were assessed as acceptable and unreasonable, and the results obtained after the adjustment are shown in Table 2. The Pareto optimality screening strategy was applied to interactively adjust the planned path, and the results obtained after adjustment are shown in Table 3. To eliminate evaluation bias caused by long time intervals, the experiment above was completed within two days.

It can be seen from Table 1, Table 2 and Table 3 that the ablation needle puncture paths automatically planned by this system are relatively reasonable. More than 50% of the automatically planned ablation needle puncture paths can be used directly. After interactive adjustment, more than 78% of the planned paths can fully meet the needs of clinicians. During the verification process, the automatic planning time starts from clicking the “Planning” button on the “Path Planning” interface and ends with the blue planned path appearing. Interactive adjustment of the puncture path time starts from clicking the “OK” button on the “Path Planning” interface and ends with a yellow planned path appearing. The automatic planning of the puncture path takes about 4 min, and the interactive adjustment of the puncture path takes about 4 min.

## 4. Discussion

We designed a liver tumor thermal ablation surgical path-planning system based on the clinical constraints of thermal ablation planning, focusing on realizing the automation of surgical path planning. Through functional verification of the system, it has been proven that the system can effectively segment key structures (liver, tumor, blood vessels, bones, skin, lungs) in CT images and use this to construct an anatomical scene of the patient’s abdominal cavity to achieve 3D visualization. In addition, based on the automatic planning method of the ablation needle puncture path proposed, the system can perform automatic planning and interactive adjustment of the ablation needle puncture path, and provide real-time feedback on the planning parameters of the puncture path.

Through the verification of the effectiveness of the system, it was proved that more than 50% of the automatically planned ablation needle puncture paths can be directly used in clinical practice. After interactive adjustment of the remaining automatically planned ablation needle puncture paths, more than 72% of the planned paths can fully meet the needs of clinicians. The reason why it is necessary to interactively adjust the partially automatically planned ablation needle puncture path is not because the puncture path does not meet the clinical multiple constraints considered by the algorithm, but because the path-planning method implemented does not fully consider the actual shape of the tumor, only abstracting the tumor as a puncture target, that is, there is a lack of relevant measures for the conformal coverage constraints of the tumor. In addition, clinicians suggest that the system can automatically plan 2–3 ablation needle puncture paths for clinicians to choose, which can avoid the problem of ablation needle puncture path planning falling into local optimality and reduce the need for interactive adjustment of ablation needle puncture paths, further reducing the time consumption of system planning ablation needle puncture paths (especially in the interactive adjustment stage).

Based on the limitations of the current work completed, follow-up research will focus on the following aspects:

(1) The system cannot realize the 3D automatic/semi-automatic segmentation function of all the key abdominal structures. It lacks the realization of segmentation methods, such as the stomach, kidney, spleen, heart, and gallbladder (bile duct). Implementing segmentation of all the key abdominal structures can improve the effectiveness of path planning.
(2) The system is mainly aimed at path planning of smaller tumors, so it does not include modules such as the prediction of the ablation coagulation area and the setting of treatment parameters. For the treatment of larger and irregularly shaped tumors, predicting the coagulation zone and selecting treatment parameters are even more important.
(3) The path planning does not consider the displacement and deformation of the liver caused by respiratory movement, does not include the consideration of the best needle insertion timing during the respiratory cycle, and does not formulate an effective needle insertion–force feedback model. Path planning based on these factors has more clinical value and is worth further research.

## 5. Conclusions

We developed a CT-guided liver tumor thermal ablation surgical path-planning system design for liver tumors with a diameter of less than 3 cm. The system achieves three functional requirements, including segmentation and 3D reconstruction of key abdominal structures in clinical CT images, automatic planning and interactive adjustment of surgical paths, and image information management. The main interface of this system is simple and easy to use. The design principle is to reduce interactive operations as much as possible and provide automated solutions. After being operated by clinicians, it is believed that it conforms to the clinical treatment process and usage habits, the planning path has high effectiveness, and the planning efficiency is better than manual planning by clinicians. It can provide assistance and guidance to clinicians in the surgical planning stage. In the future, we plan to segment all the organs in the abdominal cavity and consider respiratory movement and liver displacement and deformation caused by the ablation needle to further improve the accuracy of path planning. In addition, another research branch of the laboratory will be integrated into the system to provide a more comprehensive preoperative plan for large tumors (>3 cm) by predicting the ablation coagulation zone and setting treatment parameters. 

## Figures and Tables

**Table 1:** Effectiveness evaluation of ablation needle puncture path-planning system.

Participants	Excellent	Acceptable	Uncertain	Unreasonable
Clinician A	11	6	0	1
Clinician B	9	8	0	1

**Table 2:** Effectiveness evaluation of ablation needle puncture path-planning system (interactive adjustment).

Participants	Excellent	Acceptable	Uncertain	Unreasonable
Clinician A	14	4	0	0
Clinician B	13	5	0	0

**Table 3:** Effectiveness evaluation of Pareto optimality screening strategy for interactive adjustment of planning paths.

Participants	Excellent	Acceptable	Uncertain	Unreasonable
Clinician A	16	2	0	0
Clinician B	14	4	0	0

## Data Availability

The raw data supporting the conclusions of this article may be provided upon reasonable requests for scientific research purposes.
